# Expression of Cucumber mosaic virus suppressor 2b alters FWA methylation and its siRNA accumulation in *Arabidopsis thaliana*

**DOI:** 10.1242/bio.017244

**Published:** 2016-09-22

**Authors:** Sadia Hamera, Youngsheng Yan, Xiaoguang Song, Safee Ullah Chaudhary, Iram Murtaza, Lei Su, Muhammad Tariq, Xiaoying Chen, Rongxiang Fang

**Affiliations:** 1 SBA School of Science and Engineering, Lahore University of Management Sciences (LUMS), DHA, Lahore 54792, Pakistan; 2 State Key Laboratory of Plant Genomics and National Center for Plant Gene Research, Institute of Microbiology, Chinese Academy of Sciences, Beijing 100101, China; 3 Department of Biochemistry, Quaid i Azam University, Islamabad 54320, Pakistan

**Keywords:** Cucumber mosaic virus, CMV2b, AGO4, RdDM, FWA

## Abstract

The Cucumber mosaic virus (CMV) suppressor 2b co-localizes with AGO4 in cytoplasmic and nuclear fractions of *Arabidopsis thaliana*. Biochemical fractionation of *A. thaliana* cellular extracts revealed that 2b and AGO4 coexist in multiple size exclusions. 2b transgenic *A. thaliana* exhibited an enhanced accumulation of 24nt siRNAs from flowering wageningen (FWA) and other heterochromatic loci. These plants also exhibited hypo-methylation of an endogenous- as well as transgene-FWA promoter at non-CG sites. In corroboration, both transgenic 2b and CMV infection affected the regulation of transposons which mimics the *ago4* phenotype. In conclusion, 2b perturbs plant defense by interfering with AGO4-regulated transcriptional gene silencing.

## Abstract (precis)

**Summary:** CMV suppressor 2b perturbs host defense by interrupting epigenetic signaling. 2b causes FWA siRNAs sequestration and hypo-methylation of FWA promoter region. It shines light on a hitherto little understood mechanism of viral suppression of host epigenetics.

## INTRODUCTION

RNA silencing is a conserved nucleotide sequence-specific gene inactivation mechanism which controls a wide range of functions including gene regulation, heterochromatin formation (Matzke et al., 2004) and antiviral resistance (Ding and Voinnet, 2007). It invariably relies on a set of core elements that triggers the processing of double-stranded RNA into small RNA (sRNA) duplexes of 21-24nt in length. These sRNAs organize the complex mechanism of gene regulation at transcriptional, post-transcriptional and translational levels (Hammond, 2005). siRNA-mediated transcriptional gene silencing is not only conserved at promoter regions, but also implies histone methylation especially at the centromeric regions (Henderson and Jacobsen, 2007; Matzke et al., 2009; Mlotshwa et al., 2010). In eukaryotes, major protein families involved in RNA silencing at the transcriptional level are DCL3, RDR2, AGO4 and DNA-dependent RNA polymerases, i.e. POLIV and POLV.

In plants DCL3 is destined to produce 24nt siRNAs from transgene, repeat-associated and transposon-related sequences (Xie et al., 2004; Pontes et al., 2006). RDR2 converts target mRNA into dsRNA by the coordinated action of POLIV, and the dsRNA subsequently becomes the substrate of DCL3 to produce the secondary siRNAs (Daxinger et al., 2009). These 24nt siRNAs also called as hcsiRNAs (heterochromatin-associated small interfering RNAs), and act as mobile silencing signals to induce epigenetic changes in the heterochromatin formation by loading into AGO4 (Molnar et al., 2011). AGO4 is one of the ten AGOs in *Arabidopsis thaliana*, and is responsible for binding with the siRNAs from repeat-associated and heterochromatin-related loci; its mutant phenotype represents the loss of epigenetic modifications at several chromosomal loci including the epi-alleles FWA and Superman (Zilberman et al., 2003; Li et al., 2006; Qi et al., 2006). Similar to AGO1, AGO4 causes target transcript cleavage through its catalytic triad motif Asp-Asp-His (DDH) (Baumberger and Baulcombe, 2005; Qi et al., 2006). AGO4 mutant phenotypes show a down-regulation of 24nt siRNA accumulation and hypo-methylation of several repeats, transposons and retrotransposons including Rep2, MeaIsr, Mu1 and 5SrDNA (Qi et al., 2006), interestingly, activation of certain transposable elements was also observed (Pontes et al., 2009). Mutations in the AGO4 upstream loci DCL3 and RDR2 reduce AGO4 protein accumulation while downstream loci RNA PolV have no effect on AGO4 stability, indicating the role of siRNAs in AGO4 stability (Li et al., 2006; Havecker et al., 2010). The *ago4-1* shows a late-flowering phenotype and hypo-methylation of the imprinted gene *FWA* in *A. thaliana*. FWA methylation depends on siRNAs produced from its promoter containing short interspersed nuclear element (SINE)-related tandem repeats (Chan et al., 2004; Lippman et al., 2004).

AGO6 and AGO9 can complement the functions of AGO4 to an extent (Zheng et al., 2007; Havecker et al., 2010), but only AGO4 resists the pathogenic effects of *Pseudomonas syringae* in *A. thaliana* (Agorio and Vera, 2007). Similarly AGO1 and AGO2 have been shown to load with the Cucumber mosaic virus (CMV) siRNAs, implying the coordinated action of AGO's to direct antiviral defense (Zhang et al., 2006; Harvey et al., 2011). To combat antiviral silencing, plant viruses encode for silencing suppressors (VSRs). Often these VSRs act through sequestering the siRNAs, for instance P19 of Tomato bushy stunt virus (TBSV), HcPro of Potyvirus and 2b from CMV sequester micro RNAs and siRNAs (Lakatos et al., 2006; Duan et al., 2012); however, other VSRs adopt more complicated strategies. For example, P25 of Potato virus X and P0 of Polerovirus cause AGO1 degradation through the proteasome pathway (Bortolamiol et al., 2007; Chiu et al., 2010; Csorba et al., 2010), but the CMV 2b binds directly with the PAZ and PIWI domains of AGO1 and AGO4 and perturbs their slicer activity and accumulation of related sRNAs (Zhang et al., 2006; Gonzalez et al., 2010; Duan et al., 2012; Hamera et al., 2012). This results in the malformation of RNA silencing pathways. Interestingly, 2b was also found to downregulate the AGO4 transcript level in *Nicotiana* and *Solanum* spp. (Cillo et al., 2009; Ye et al., 2009) implying its role in the transcriptional gene silencing (TGS) pathway across the plant species.

In this study we found that AGO4 accumulation is not effected by 2b and CMV infection in *A. thaliana* plants; however overlapping expression patterns in cellular fractions and size exclusion chromatography (SEC) shows the ability of 2b to interact with AGO4. Moreover, 2b perturbs FWA-related siRNA accumulation which results in FWA hypo-methylation at non-CpG sites thus supporting multiple functions of 2b in conjunction with AGO4.

## RESULTS

### CMV infection and transgenic expression of 2b induces phenotypic anomalies in *A. thaliana*

CMV-induced variable symptom severity in host plants is correlated with the virus strains. Subgroup IA and IB strains show higher virus load in host plants and induce more abrogating phenotype and RNA silencing suppression compared with subgroup II strains (Zhang et al., 2006; Lewsey et al., 2007). Considering that symptom induction is related to silencing suppressor protein 2b accumulation (Zhang et al., 2006), we sought to address 2b-related phenotype variations in the host plant. We transformed the 2b gene from the CMV subgroup IB strain Shandong (SD), into *A. thaliana* ecotype Columbia-0 (Col-0). The 2b gene was engineered so that the resulting protein was tagged with myc or mycHis (mh), while the P19 gene from TBSV was engineered such that P19 was tagged with mh and used as a control.

**Fig. 1.**
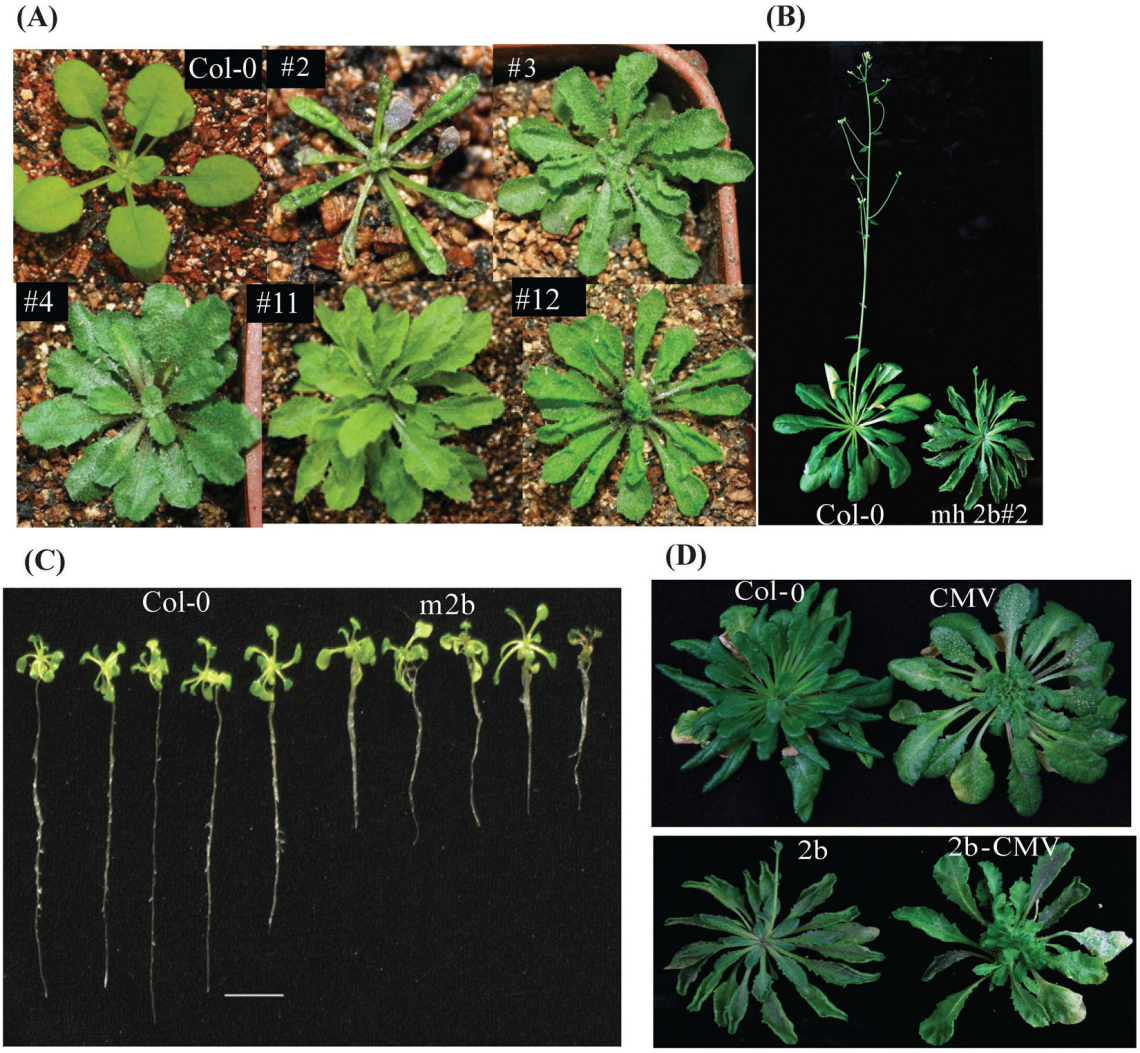
**CMV 2b causes phenotypic anomalies in *A. thaliana***. (A) mychis 2b (mh 2b) transgenic Col-0 represent elongated, upward curled and strongly serrated leaves. (B) 2b transgenic line mh 2b#2 flowering time compared with control Col-0. Photographs were taken 4-5 weeks after transferring to soil. (C) Root growth impaired in 2b transgenic plants. 16-day-old seedlings on half MS medium representing shorter root length compared with control Col-0. (D) *A. thaliana* Col-0 and 2b transgenic mh 2b#2 line representing phenotype after CMV infection. 2b#2 exhibited more phenotypic anomalies compared with alone 2b transgene or CMV infection. Plants were grown under short day conditions.

The 2b transgene expression induced various phenotypic anomalies from mild to severe in transgenic *A. thaliana* lines. Aerial parts of severely symptomatic plants from 2b transgenic lines showed elongated, narrowed, upward-curled and serrated leaves while plants with milder symptoms showed strongly serrated and curled leaves. About 30% of the lines with severe symptoms were either infertile or died shortly after the emergence of true leaves. Interestingly, some of the lines were significantly late flowering compared with the wild-type Col-0 and less severe lines (Fig. 1A,B).

Similarly there was notable growth retardation in the roots of plants from the 2b transgenic lines compared with that of Col-0 (Fig. 1C). Interestingly, when plants that were transgenic for 2b were infected with CMV, they exhibited a further delay in flowering and enhanced abnormal leaf structure (Fig. 1D) implicating a role of CMV 2b in plant morphology (Praveen et al., 2008).

**Fig. 2.**
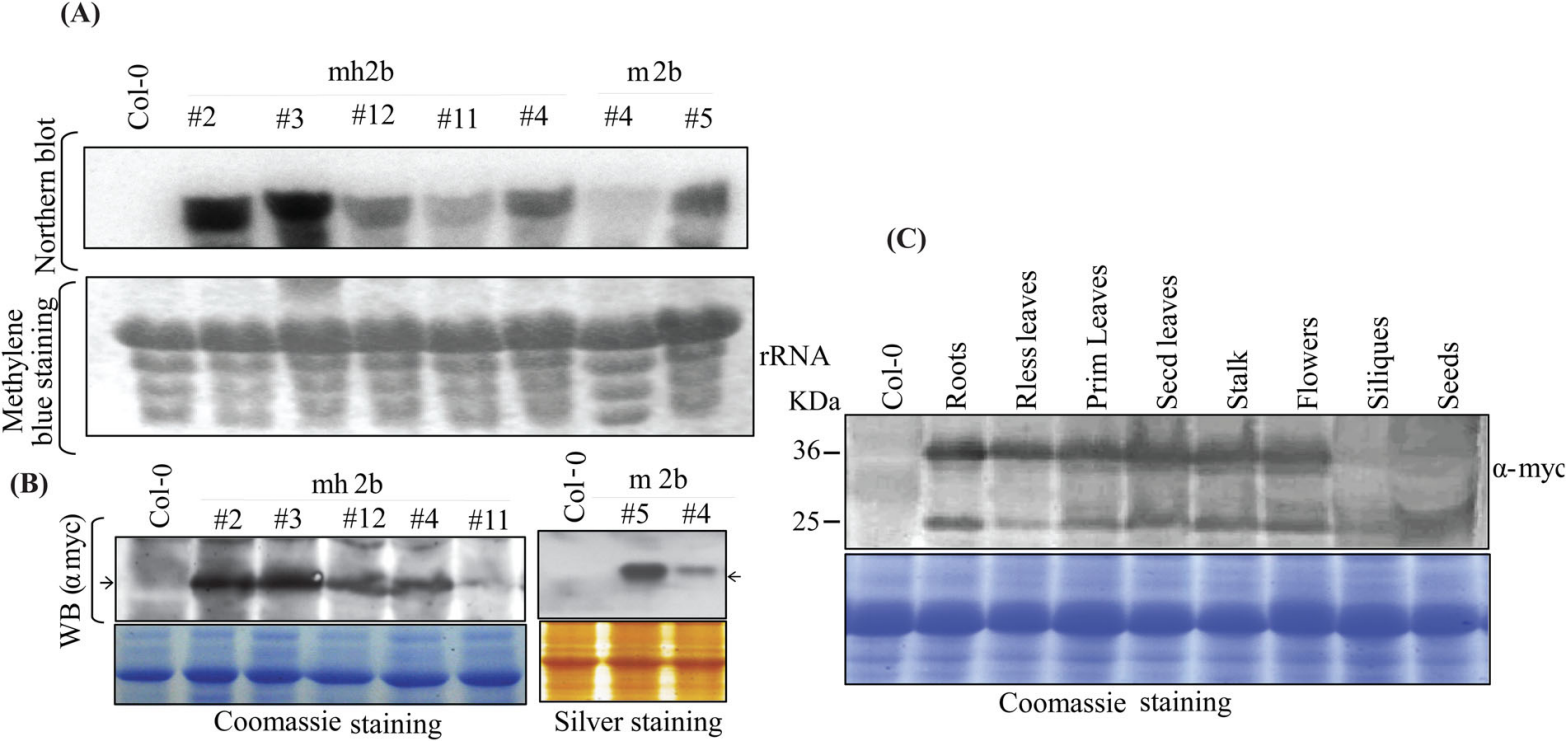
**CMV 2b transcript and protein accumulation in transgenic plants.** CMV 2b (SD strain) transcript and protein levels in 2b transgenic *A. thaliana* Col-0 plants. Transgenic plant numbers are given on top of panels. (A) 2b mRNA detected through northern blot, 2b-UTR sequence used as probe for hybridization. Membrane stained with methylene blue prior to northern blot, representing rRNA, was used as loading control. (B) Polyclonal myc antibody was used for detection of 2b in immunoblot. Coomassie blue and silver stained gels served as loading controls for the respective samples. Arrowheads on both right and left side of blots are representing 36 kDa marker position. (C) 2b expression pattern in different plant tissues. Protein samples were prepared by boiling plant lysates in extraction buffer which contains both soluble and insoluble forms of 2b. 2b transgenic lines #2, #3 and #4 were checked for the expression study however data from #2 is presented in this figure. Rless (rootless leaves) collected from MS medium plates after 14 days of sowing, Prim (Primary) leaves collected from plants 10 days post-transfer to soil, Secd (secondary) leaves collected 20 days post-transfer to soil.

A general concomitance was observed between 2b-induced phenotypic anomalies and 2b protein accumulation (as well as 2b's transcript) in *A. thaliana* (Fig. 2A). Examination of 2b protein accumulation in different tissues showed significant accumulation of 2b in different tissues including leaves, stalk, flower and roots (Fig. 2C). Overall 2b was found in all plant tissues tested, and exhibited an association with the late-flowering and abrogate phenotype of *A. thaliana*.

### Ubiquitous expression of the 2b-AGO4 complex in *A. thaliana*

**Fig. 3.**
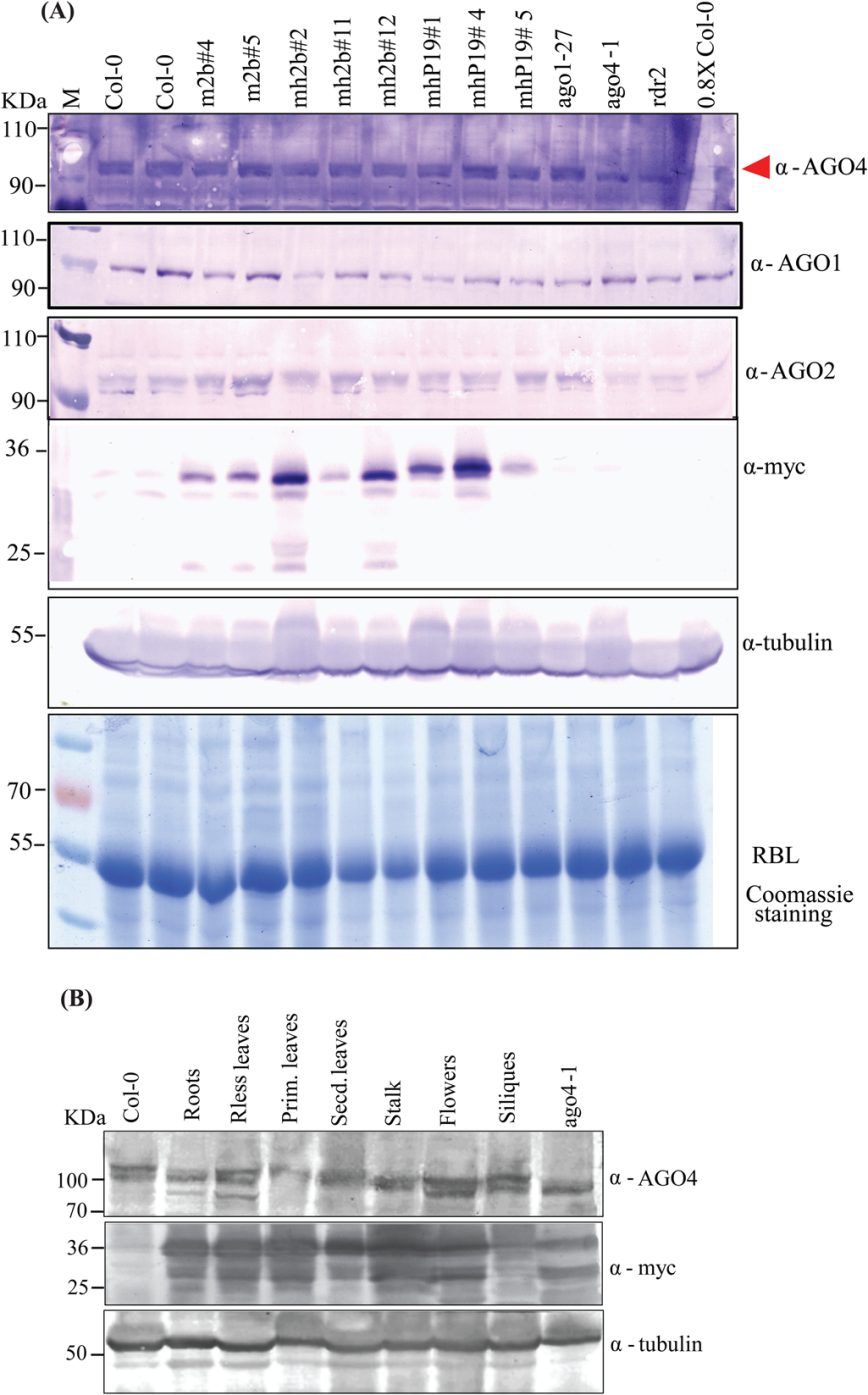
***A. thaliana* Argonaut4 (AGO4) protein expression in 2b transgenic lines.** (A) Leaf samples were collected from 20-day-old *A. thaliana* seedlings. 2b transgenic line numbers are represented on top of panels. Protein samples were immunoprobed with anti-AGO4 antibodies (top panel), anti-AGO1 (2nd panel), anti-AGO2 (3rd panel) and anti-myc (4th panel). Anti-myc antibodies used for detection of 2b and P19 protein accumulation. *ago1*, *ago4* and *rdr2* mutants were used as controls. 0.8xCol-0 represents 1/8th loading compared with rest of the samples. Tubulin was used as internal control for western blot. Coomassie blue staining of the large subunit of RUBISCO (RbL) served as a protein loading control (bottom panel). Arrowhead indicates the upper band for AGO4 protein accumulation in respective samples. (B) AGO4 protein expression in different tissues of 2b transgenic plants. AGO4 antibody typifies two bands, where the lower band is considered as cross-reacting and upper band is for AGO4. *ago4-1* served as control, representing lower band. Tubulin served as loading control.

2b is known to bind with RISC protein AGO4 and its associated siRNAs, and this induces loss of AGO4 slicer activity (Zhang et al., 2006; Hamera et al., 2012). However, the biochemical association between 2b and AGO4 and the role of siRNAs in this interaction is not clear. Having found an association between symptom induction and 2b protein accumulation in different tissues (Figs 1 and 2), we investigated the *in vivo* interaction between 2b and AGO4. For that, we studied the possible effects of 2b on the AGO4 transcript and protein accumulation in 2b transgenic and CMV infected *A. thaliana*, respectively. No variation was observed in AGO4 mRNA accumulation (Fig. S1A). Similarly, no significant difference was observed between AGO4 protein accumulation in 2b transgenic plants and control; however, there is a possibility of AGO1 protein suppression in some 2b transgenic lines (Fig. 3A). In addition to AGO4 protein expression, our reporter-based assay also exhibited comparable AGO4 promoter-derived GFP expression in both Col-0 and 2b (Fny strain) transgenic plants. This provided further credence to 2b's inability to suppress AGO4 *in planta* (Fig. S1B,C).

2b is reported to have an interaction with AGO4 (Hamera et al., 2012). To investigate the specific role of AGO4 in TGS and immunity, we evaluated its tissue-specific expression in 2b transgenic plants. AGO4 accumulation was observed in all tissues including roots, stalk, leaves, flowers and siliques (Fig. 3B).

### 2b-AGO4 pervasive existence in cellular fractions

**Fig. 4.**
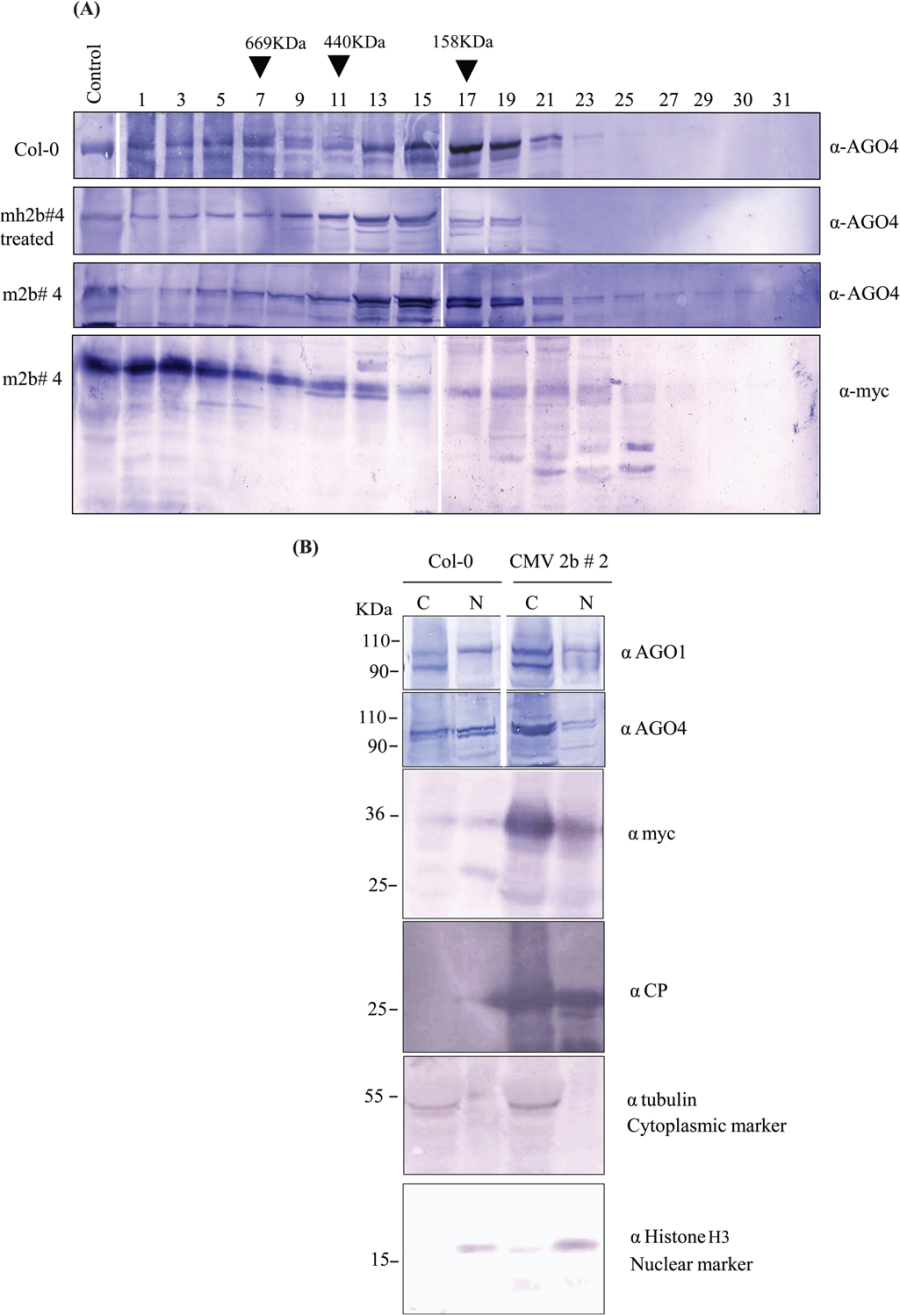
**2b-AGO4 pervasively exist in cellular fractions.** (A) 2b co-distributes with AGO4 in high molecular weight fractions. Col-0 and 2b transgenic m2b#4 plants protein lysate fractions were eluted by SEC and analyzed through western blot. 2b co-distributed significantly with AGO4 (panel 2-4). Arrows indicate fractions where the protein standards peaks were examined and standard curve was drawn. Control represents the sample taken before SEC. ‘Treated’ represents sample treated with RNAse A before SEC. (B) 2b and AGO4 co-expression in cellular fractions (nuclear and cytoplasm) of Col-0 and 2b transgenic line infected with CMV. Anti-tubulin and anti-histone H3 used for cytoplasm and nuclear markers respectively. CMV coat protein (CP), nuclear (N), cytoplasm (C).

*In vivo* interaction of 2b and AGO4 is envisaged to rely on siRNAs leading to the formation of a tripartite complex, i.e. 2b-siRNA-AGO4 (Hamera et al., 2012). To ascertain this interaction, we investigated the existence of 2b-AGO4 complex *in vivo*. SEC of *A. thaliana* cellular lysates from 2b transgenic- and control-Col-0 was performed. Complexed 2b eluted at fractions of around 158 kDa to over 669 kDa (fractions 1-17) (Fig. 4A). Interestingly, the elution profile of AGO4 correlated with high to low molecular-weight 2b complexes, which included a minor fraction of AGO4-2b over 440 kDa (Fig. 4A). Similar results were obtained from the samples treated with RNaseA (Fig. 4A), thus ruling out the possible role of siRNAs in 2b and AGO4 interaction.

After observing 2b and AGO4 in the same fractions, we set out to find their co-localization in cellular compartments. 2b and AGO4 were observed in both nuclear and cytoplasmic extracts of 2b transgenic CMV-infected plants (Fig. 4B). The existence of 2b-AGO4 in nuclear and cytoplasmic fractions as well as in SEC fractions alludes to formation of convoluted complexes. Given that the AGO4-POLV (NRPE1) complex also elutes at 440 kDa, the 2b-AGO4 fraction may also have a contribution from AGO4-PolV (NRPE1) or other equally weighted AGO complexes.

### 2b causes hypo-methylation of FWA in tandem with accumulation of FWA-related 24nt-siRNAs

**Fig. 5.**
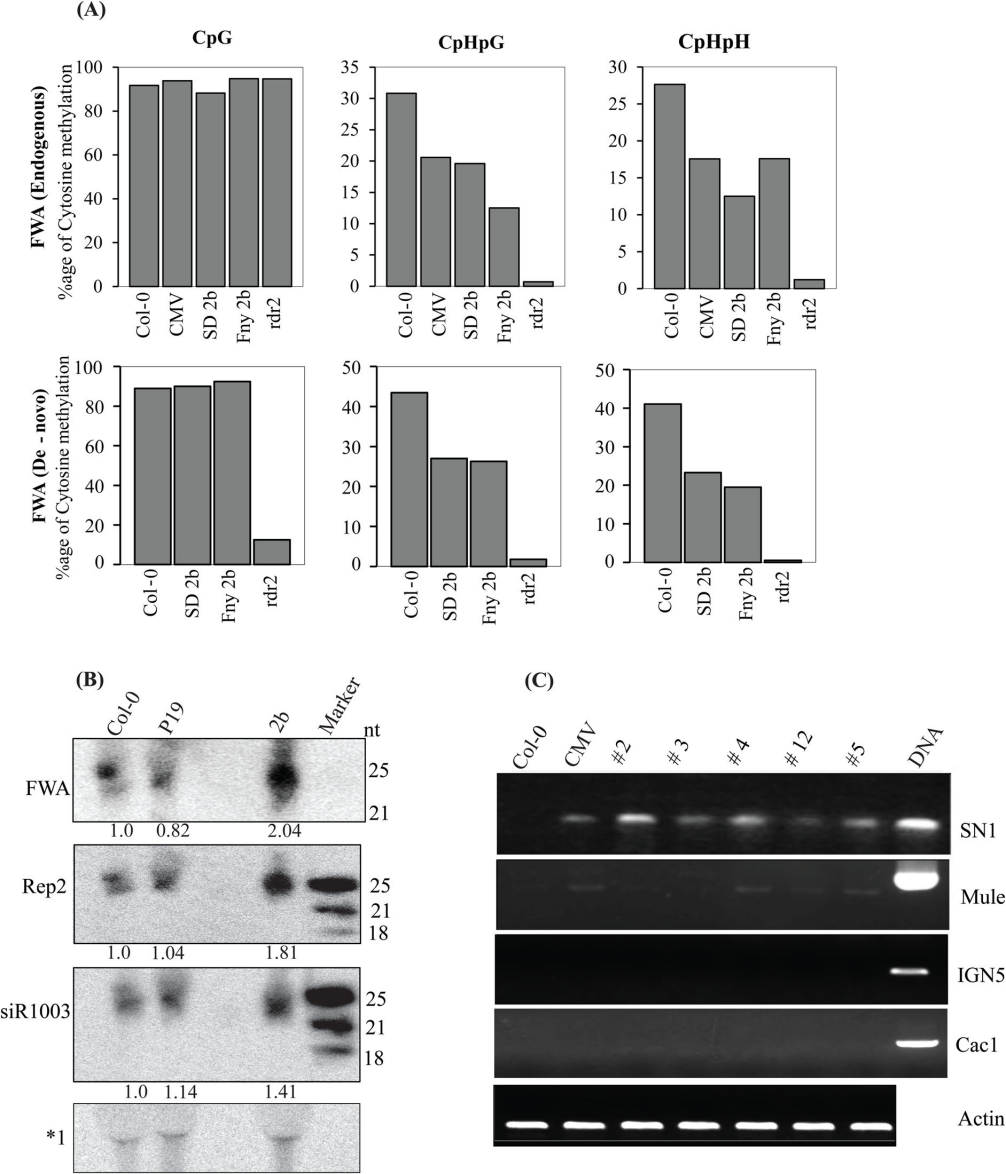
**FWA hypo-methylation and siRNAs over-accumulation in 2b transgenic lines.** (A) FWA CHG and CHH methylation was reduced in 2b transgenic and CMV-infected plants. This hypo-methylation was observed both for endogenous (upper panel) and transgene (de novo) FWA (bottom panel). *rdr2* mutant was used as control. (B) Northern blot analysis reveals 24nt FWA, Rep2 and siR1003 siRNAs over-accumulation in 2b transgenic lines compared with Col-0 and P19 lines. Nonspecific bands appearing at U6 position due to cross-reactivity of FWA LNA probe (marked as *1) further used as loading control. The numbers below each lane refer to expression levels relative to wild type. (C) Semi-quantitative RT-PCR represented increased SN1 and Mule expression in CMV-infected and 2b transgenic lines; however, transposon CAC1 and intergenic region (IGN5) accumulation was not observed. Actin served as loading control.

FWA encodes a homeodomain gene that is silenced by promoter methylation at tandem SINE repeats through siRNAs generated from them. AGO4 regulates FWA methylation while the *ago4-1* mutant blocks de novo DNA methylation of the FWA transgene (Chan et al., 2004). This results in an overexpression of the transgene, thus producing a late-flowering phenotype. Following up on reports of 2b-AGO4 mediated hypo-methylation of certain AGO4 related loci (Duan et al., 2012; Hamera et al., 2012), we investigated 2b's specific inhibitory effect on AGO4-dependent FWA methylation. 2b transgenic and CMV-infected plants were evaluated for FWA de novo and maintenance methylation. Hypo-methylation of endogenous FWA was observed at CHG and CHH sites, both in CMV-infected and 2b transgenic plants (Fig. 5A). Importantly, transgene FWA manifested loss of both CHG and asymmetric methylation (Fig. 5A, bottom panels). Additionally, flowering time and leaf number were not significantly altered as compared to *rdr2* control (data not shown). This loss of methylation was accompanied with an increased siRNA accumulation of FWA as compared with controls (Col-0 and P19) (Fig. 5B). Furthermore, elevated levels of AGO4 related siR1003 and Rep2 siRNAs were observed in 2b transgenic plants (Fig. 5B).

AGO4 is known to preferentially target non-CG methylation which is evidenced by Rep2, FWA, Superman, SN1, MeaISR and SimpleHat2 (Zilberman et al., 2003; Qi et al., 2006; Duan et al., 2012); however, *ago4-1* mutant also exhibits loss of CG methylation of transgene FWA which is necessary for its de novo silencing (Chan et al., 2004). Since AGO4 effects are loci-specific (Zilberman et al., 2003), it may be that 2b-AGO4 has a limited effect on the establishment of FWA de novo DNA methylation. Such regulatory aberration, however, cannot produce a strikingly distinct phenotype as evidenced by FWA transformation in 2b lines.

### 2b activates transposons in *A. thaliana* transgenic plants

Recruitment of 24nt siRNAs by AGO4 leads to DNA methylation of transposons and retro-transposons. A mutation in AGO4 results in loss of methylation and accumulation of transposable elements (He et al., 2009). To examine the functional loss resulting from such 2b-AGO4 interaction, we analyzed the activation of transposable elements in 2b transgenic plants. SN1 retrotransposon accumulation was observed while transposon Cac1 and intergenic region (IGN5) transcription remained unchanged. Similarly, Mule transposon exhibited slight accumulation in both CMV-infected and 2b transgenic plants (Fig. 5C). This SN1 accumulation may be attributed to hypo-methylation at CHG and asymmetric sites (Hamera et al., 2012). Taken together, transposon expression profile in 2b transgenic plants is akin to their activation in *ago4* mutants (He et al., 2009; Pontes et al., 2009).

## DISCUSSION

The CMV VSR 2b is a unique viral suppressor with multifunctional activities in virus movement, symptom induction, silencing suppresion and host genome methylation through interlocutors such as AGO1, AGO2 and AGO4 (Pumplin and Voinnet, 2013). In this study we evaluated the interactions between 2b and AGO4 followed by their downstream effects. The expression of 2b under 35S promoter is robust and ubiquitous and leads to phenotypic anomalies in leaves and roots as well as late flowering (Fig. 1). Our 2b transgenic plants report elevated expression of AGO4 protein in flowers, siliques and young leaves as compared to other plant tissue. This elevated AGO4 expression was analogous to mRNA transcript accumulation in tissues, while for control AGO2, a nonsignificant correlation was found between the mRNA and protein expression levels (Mallory and Vaucheret, 2010).

Co-expression of 2b and AGO4 attenuates the activity of AGO4 due to complex formation between the two proteins. Furthermore, transposon activation was observed in both 2b transgenic and CMV-infected plants as evidenced by SN1 accumulation (Fig. 5C), which phenocopies the transposon accumulation in *ago4* mutant (Pontes et al., 2009). Hypo-methylation of SN1 at CHG and CHH loci, and corresponding 24nt siRNAs over-accumulation in 2b transgenic lines, further exhibits the regulatory function of 2b upon AGO4 (Duan et al., 2012; Hamera et al., 2012).

Over-accumulation of 24nt FWA siRNAs in 2b transgenic lines leads to a loss of maintenance methylation at CHG and non-symmetric sites (Fig. 5A,B), providing evidence of an anomalous regulation by 2b. However, accumulation of endogenous siRNAs affected by 2b is in contrast to *ago4* mutant, which deploys 2b engendered siRNA sequestration to limit its availability to AGO4 for target loci methylation. The ability to sequester siRNA is common amongst suppressor proteins, however, the sequestered siRNA size may vary; for instance, P19 binds with 21nt miRNA duplexes while Flock house virus (FHV) B2 protein binds with longer siRNAs (Pelaez and Sanchez, 2013). This eludes 2b's role in sustaining AGO4 function endogenously. However, the establishment of FWA de novo silencing and flowering phenotypes depend upon CG methylation. This is evident in AGO4 mutants exhibiting a late-flowering phenotype due to a loss of CG methylation of the FWA transgene; however, 2b impedes the establishment and maintenance of non-CG and asymmetric methylation. The current study also complies with 2b's role in hypo-methylation of the Superman gene (Hamera et al., 2012), in which non-CG hypo-methylation exhibited no effects on the phenotype.

Moreover, Beet severe curly top virus (BSCTV) suppressor C2 is known to suppress FWA de novo methylation in the C2 transgenic plants (Zhang et al., 2011). C2 defends the virus genome by inhibiting its DNA methylation in infected plants, thus providing the virus with an opportunity to replicate freely in host cells. Methylation may act as the first line of defense against DNA viruses replicating in the nucleus, whereas RNA viruses such as CMV may encounter host defenses in the cytoplasm. Viruses residing in the cytoplasm thus avoid getting their genomes targeted by methylation but are vulnerable to other host defense mechanisms. CMV CP and 2b exist in cytoplasmic as well as nuclear fractions (Fig. 4B), signifying their dual roles in post-transcriptional gene silencing (PTGS) and transcriptional gene silencing (TGS) (Du et al., 2014). Moreover AGO4 redistribution in cytoplasm and its shuttling back to the nucleus along with hcsiRNAs describe its role in hijacking the cytoplasmic hcsiRNAs for methylation in nucleus (Ye et al., 2012). CMV can therefore be utilized as a model for eliciting viral strategies adopted to overcome host defenses.

The cytoplasmic localization of 2b may suggest its crucial role in PTGS activity, while its nuclear localization may be required for abrogating AGO4-related functions (Fig. 4B). However, the interaction of cytoplasmic 2b with hcsiRNAs and its shuttling to the nucleus along with AGO4 is not yet clear. Further research is being carried out to resolve the crosstalk between localization of 2b in different cellular compartments and the resultant influence on functional activities of AGO4. Furthermore, CMV- and 2b-mediated intervention highlights 2b's potential to evade AGO4-mounted defense mechanisms. Taken together, FWA de novo CG methylation caused by AGO4 represents a perplexing picture of AGO4's function independent of 2b hallmarks. One possible explanation could be that AGO4 effect is loci-specific, as methylation on Ta3 has been shown to remain unchanged in *ago4* mutants (Zilberman et al., 2003). It would be insightful to see 2b's effect on less-complicated non-CG methylation-dependent loci such as SDC in 2b transgenic plants (Henderson and Jacobsen, 2008).

## MATERIALS AND METHODS

### DNA constructs and plant transformation

For plant transformation, CMV 2b (SD strain) with its 3′ UTR and TBSV P19 were cloned into pBA002 vector (*SmaI-SpeI*). pBA002 vector is constructed with CaMV 35S promoter, myc tag and myc-His (mh) tags as described previously (Hamera et al., 2012). Transformation was performed by floral dip method, and the positive transgenic plants were further tested for protein expression and phenotype.

### Plant materials and growth conditions

For plant growth, seeds were sterilized with 30% bleach for about 7 min and neutralized with repeated washes of distilled sterilized water. Sterilized seeds were spread on MS plates and stratified at 4°C for 2-3 days before transferring to 22°C (16 h light:8 h dark) greenhouse conditions. 14-day-old plants were transferred to soil, and allowed to grow under controlled conditions.

### Western blotting

Total proteins were extracted from plants following the method described by Hamera et al. (2012). Supernatant containing soluble proteins and plant lysate's boiling in protein loading dye lead to formation of both soluble and insoluble fractions. These fractions were separated by western blot. Protein samples from Col-0 and transgenic plants were first quantified through nano-drop and Coomassie staining then subjected to western blot. Anti-myc antibody (1:10,000 dilution, cat no. A00704, GenScript) while anti-AGO1, anti-AGO2 and anti-AGO4 (1:1000 dilution) were used for western blotting. AGO1, AGO2 and AGO4 antibodies were mcklh conjugated and raised against VRKRRTDAPSEGGEGC, NRGQGRGEQQC and CRELKKRNPNENGEFE peptides (Havecker et al., 2010) respectively, from Genscript (www.genscript.com). Tubulin (Abcam-ab6046) and anti-histone H3 (Abcam-ab1791) were commercially purchased from Abcam while anti CMV CP was previously generated by our lab from anti-rabbit serum.

### DNA isolation and bisulfite sequencing

*A. thaliana* genomic DNA was extracted from leaves using a Wizard Genomic DNA purification kit (Promega). Bisulfite conversion was done with EZ DNA Methylation-Gold kit (ZYMO Research) according to the manufacturer's instructions. Bisulfite-converted DNA samples were used for FWA template amplification with Platinum Taq Polymerase (Invitrogen). Primers are listed in Table S1. The PCR products were cloned into pGEM-T easy vector (Promega). For each sample, about 12-15 individual clones were sequenced. PCR conditions were 95°C-5 min (1×), 95°C-30 s, 68°C-30 s, 72°C for 70 s (6×), 95°C-30 s, 54°C-30 s,72°C-70 s (30×) following 72°C-5 min and standby at 4°C. The cytosine methylation analysis was done using CyMATE program (www.cymate.org).

### RNA extraction and northern blotting

Total RNAs were extracted from either leaf tissues or flowers, using Trizol (Invitrogen). Small RNA enrichment was done with 4 M LiCl. Briefly LiCl addition was followed by incubation at −20°C for 3 h. Supernatants containing sRNA were taken after brief centrifugation at 12,000 ***g*** for 5 min. 1× isopropanol was added into supernatant and centrifuged at high speed for 30 min. sRNA pallets were washed once with 75% ethanol. Isolated sRNAs were dissolved in 50% formamide. Enriched sRNA concentrations were measured with nano-drop (Thermo, USA) while purity from large molecular weight RNA fractions was determined by running samples on 2% agarose gels. For northern blotting, sRNA samples were run on 17% Urea-PAGE gel and transferred to Hybond N^+^ membrane (Amersham). Single stranded miRNA marker (Biolabs) was run in parallel to determine the size of sRNAs on the blot. sRNA probes as described in Table S1 were end labeled with T4PNK (Biolabs). Blots were used to rehybridize after stripping off the previous one. Hybridization signals were detected by phosphor imager (GE Healthcare Life Sciences) or autoradiography. For mRNA northern blot, samples were transferred from agarose-mops gel onto nylon membrane and hybridized with α-p^32^labeled 2b-3′UTR probe.

### RT-PCR and quantitative real-time PCR analysis

Total RNAs were treated with DNaseI (Takara) and used for cDNA synthesis. cDNA synthesis was carried with Superscript III reverse transcriptase (Invitrogen) using oligodT primers according to the manufacturer's instructions. Real-time PCR was performed using SYBR Green (Genscript) and primers are listed in Table S1. Actin was used as an internal control.

### Virus infection assay

For virus infection samples were virus-sap rub inoculated. Briefly pre-infected frozen plant tissues were ground in cold 5 mM NaHPO_4_ (pH 7.4) buffer. Virus particles containing buffer were rubbed three times on each of the 3-4 top leaves of the plant. Before inoculation, leaves were sprayed with carborundum to produce friction for rubbing. Carborundum produces heat, so after about 2-3 h of rubbing, plants were sprayed with water and covered to keep moist. Samples were collected at 14 days of post infection.

### Gel filtration assay

Whole plant protein lysates were prepared from three to four weeks old wild-type Col-0 and 2b transgenic plants. Protein extracts were filtered through a 0.2 µm filter after high speed centrifugation. Size exclusion was done by a Superdex-200 gel filtration column as described previously (Li et al., 2006). For size determination, a standard curve was generated using the calibration proteins thyroglobulin (669 kDa), ferritin (440 kDa), aldo (158 kDa), conal (77 kDa) and oval (15.49 kDa).
